# A new implementation of high-throughput five-dimensional clone pooling strategy for BAC library screening

**DOI:** 10.1186/1471-2164-11-692

**Published:** 2010-12-06

**Authors:** Frank M You, Ming-Cheng Luo, Kenong Xu, Karin R Deal, Olin D Anderson, Jan Dvorak

**Affiliations:** 1 Department of Plant Sciences, University of California, Davis, CA, 95516, USA; 2 Genomics and Gene Discovery Research Unit, USDA-ARS, Western Regional Research Center, Albany, CA, 94710, USA

## Abstract

**Background:**

A five-dimensional (5-D) clone pooling strategy for screening of bacterial artificial chromosome (BAC) clones with molecular markers utilizing highly-parallel Illumina GoldenGate assays and PCR facilitates high-throughput BAC clone and BAC contig anchoring on a genetic map. However, this strategy occasionally needs manual PCR to deconvolute pools and identify truly positive clones.

**Results:**

A new implementation is reported here for our previously reported clone pooling strategy. Row and column pools of BAC clones are divided into sub-pools with 1~2× genome coverage. All BAC pools are screened with Illumina's GoldenGate assay and the BAC pools are deconvoluted to identify individual positive clones. Putative positive BAC clones are then further analyzed to find positive clones on the basis of them being neighbours in a contig. An exhaustive search or brute force algorithm was designed for this deconvolution and integrated into a newly developed software tool, FPCBrowser, for analyzing clone pooling data. This algorithm was used with empirical data for 55 Illumina GoldenGate SNP assays detecting SNP markers mapped on *Aegilops tauschii *chromosome 2D and *Ae. tauschii *contig maps. Clones in single contigs were successfully assigned to 48 (87%) specific SNP markers on the map with 91% precision.

**Conclusion:**

A new implementation of 5-D BAC clone pooling strategy employing both GoldenGate assay screening and assembled BAC contigs is shown here to be a high-throughput, low cost, rapid, and feasible approach to screening BAC libraries and anchoring BAC clones and contigs on genetic maps. The software FPCBrowser with the integrated clone deconvolution algorithm has been developed and is downloadable at http://avena.pw.usda.gov/wheatD/fpcbrowser.shtml.

## Background

Integrated genetic and physical maps are extremely valuable resources for map-based gene cloning, comparative genome analysis, and sequencing and assembly of large and complex genomes. Screening bacterial artificial chromosome (BAC) libraries is an indispensable step for integration of genetic and physical maps, by which BAC clones and contigs can be placed and ordered on a genetic map. The use of an appropriate BAC pooling strategy [1,2] maximizes work efficiency. Two different approaches, hybridization-based and PCR-based, are available for BAC library screening. The hybridization-based approach is based on multi-dimensional pools of molecular markers or probes hybridizing with high-density BAC library screening membranes to identify the BAC clones associated with specific nucleotide sequences or genes. Overgo probes have been used for large scale physical mapping of plant and animal genomes, such as those of soybean [3], maize [4] and human [5]. Because a single overgo probe may hybridize with clones in multiple contigs and several probes may hybridize to the same clone, the overgo probes often fail to unequivocally associate a contig with a locus on a genetic map. This may arise from gene duplication, repeat sequences in a BAC clone or probe, or false positives.

A PCR-based six dimensional (6-D) BAC clone pooling strategy has been successfully used by other groups for BAC library screening in sorghum [6], maize [7] and soybean [8]. This pooling strategy includes a conventional three-dimensional (3-D) stack, called plate pool, face pool and side pool, and an additional three types of pools, called row pool, column pool and diagonal pool. A positive clone in a BAC plate is located by only three types of pools; the other three types of pools are used only for verification. Hence, this strategy not only uniquely defines individual clones and efficiently eliminates false positives but also reduces the tedious task of individual clone verification. However, the six dimensions result in a large number of pools for PCR screening, and limit the size of BAC libraries to be screened. For example, a total of 184 pools were generated for 24, 576 BAC clones (~4× genome equivalent) in sorghum [6], 288 pools for 110,592 BAC clones (~6× genome equivalent) in maize [7], and 208 pools for 49,152 BAC clones (~6.6× genome equivalent) in soybean [8]. Because the number of pools that must be screened is a function of the BAC library size, the workload for screening larger BAC libraries, such as those of the grasses in the tribe Triticeae that includes wheat, barley and rye, will be unacceptably high. For example, in the physical mapping project (http://wheatdb.ucdavis.edu) of *Aegilops tauschii*, the diploid ancestor of the wheat (*Triticum aestivum*) D genome, five BAC libraries comprise a total of 302,976 clones (in 789 384-well plates), which equal to ~8.5× *Ae. tauschii *genome equivalents. If the same pooling strategy as had been used in maize were used, 454 pools (131 × 48 × 48 for the basic 3-D stacks plus three additional dimensions with an equal number of pools) would be needed. This would be extremely laborious in DNA pool preparation and PCR screening. In order to reduce pool number and relieve PCR workload, Luo et al. (2009) [9] evaluated a high-throughput five-dimensional (5-D) clone pooling strategy based on both Illumina's GoldenGate assay and PCR screening of *Ae. tauschii *BAC clones. The major points of this strategy include: (1) Conventional 3-D grid design (plate, row, and column pools corresponding to plate, face, and side pools respectively in the 6-D strategy [6-8]) with the plate pools further grouped into two-dimensional (2-D) pools, referred to as super pools in Luo et al. 2009 [9]. Because the pooling procedure involves five different DNA pool sets, this design was called a 5-D clone pooling strategy [9]. (2) Super pools screened by Illumina's GoldenGate assay and row and column pools screened by PCR. (3) Positive plate pools at 3-D intersections further verified by PCR to find positive plate pools among the putative positives. This strategy reduces pool number and adopts the highly parallel GoldenGate assay for clone screening, and makes high-throughput clone screening possible for large genomes. With this strategy, 95% of Illumina's GoldenGate EST-based oligonucleotide markers unequivocally assigned BAC clones to loci on the genetic map [9].

The 5-D clone pooling strategy still requires a considerable effort to identify positive plates among the putative positive plates in super pool screening by PCR assays. In addition, clone row pools and column pools also need to be screened by PCR. Here we describe a new implementation of this strategy, in which clone row and column pools are further divided into sub-pools with 1~2× genome coverage to minimize the number of positive pools among the putative positive pools. The rationale for limiting the number of genome equivalents screened at a single time is that the number of false positives increases exponentially with the number of genome equivalents screened. If a 1× genome equivalent is screened, then there is an average of one plate row and plate column intersection and hence no false positive plate. If a 2× genome equivalent is screened, then there are on average four plate row and plate column intersections with four putative positive plates, of which two are false positive. If a 3× genome equivalent is screened, then there are on average nine plate row and plate column intersections with nine putative positive plates, of which six are false positive.

In the implementation of the screening strategy pursued here, all pools are screened with the Illumina GoldenGate assays. The assay data is then combined with BAC contig data and overlapping clones in BAC contigs are employed as additional information to discriminate between pool intersections that harbour positive clones and intersections that are false positive. A computational algorithm was designed for this implementation of the 5-D pooling strategy, which was integrated into a newly developed software tool, FPCBrowser, for analyzing pooling data.

## Results

### Clone deconvolution algorithm

**Figure 1:**
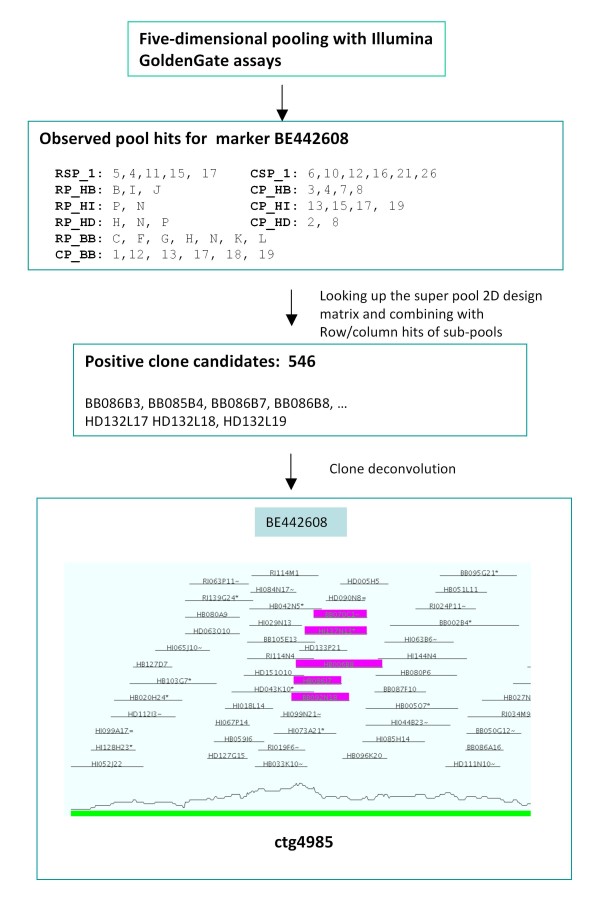
**Schematic display of the deconvolution of 5-D clone pools with the Illumina GoldenGate assay and a contig map**. A total of 302,976 BAC clones of *Ae. tauschii *were screened for the SNP marker BE442608. In the super pool screening step, 5 row hits and 6 column hits were observed and thus 30 potential plate hits were obtained. To screen plate rows and plate columns, the entire stack of plates was split into 5 smaller stacks (sub-pools) with only 1~2× coverage based on individual BAC libraries (see Table 2) and those sub-pools (RP_HB and CP_HB, RP_HI and CP_HI, RP_HD and CP_HD, and RP_BB and CP_BB) were screened separately, dramatically decreasing the number of candidate positive clones and F+ clones. By combining observed plate hits and row/column hits for each sub-pools, a total of 546 candidate clones (144, 24, 42 and 336 candidate clones for HB, HI, HD and BB sub-pools, respectively) were obtained. Of the candidate clones, 362 were located in 336 contigs, 336 clones were not included in contig assembly because of substandard or failed fingerprints, and the remaining 23 clones were singletons. The deconvolution algorithm was used to detect truly positive (TP) clones among the 362 candidate clones. Five TP clones, BB092N18, BB070C1, HB086J7, HI137N13, and HB006B8 were detected in ctg4985. The SNP marker derived from EST BE442608 was previously mapped on the genetic map of *Ae. tauschii *chromosome 2D, hence contig ctg4985 was anchored at this locus on the 2D genetic map. This inference agrees with the previous anchoring of contig ctg4985 [9].

Clone deconvolution identifies clone(s) that have a desired marker by analyzing information about pool intersections in a multi-dimensional BAC pooling design. The 5-D pooling strategy employing row-pools (RP), column-pools (CP), plate pools (PP), column super-pools (CSP), and row super-pools (RSP) is graphically detailed in Luo et al. 2009 [9]. The 5-D deconvolution algorithm is based on information generated by a combination of pool screening with the Illumina GoldenGate assay and clone overlaps in the contig map (See details in **Methods**). The Illumina GoldenGate assay detects a positive plate RSP, plate CSP, clone RP and clone CP for each molecular marker (Figure 1). A positive plate pool is at the intersection of plate RSP and plate CSP in the super pool 2-D design matrix (see Table S1 in Additional file 1). Candidates for positive clones are at intersections of positive clone RPs, and positive clone CPs, and positive plate pools. In each set of positive clone candidates only a few clones are truly positive (TP) clones; the rest are false positive clones at multiple intersections generated when more than a single positive RP and CP are obtained by Illumina GoldenGate screening. Since multiple genome equivalents are always screened, more than one TP clone is expected. Because TP clones share the same marker, they should share also a portion of their fingerprint profiles and hence be neighbours in a contig (unless specified, it is assumed throughout that contig assembly generates only "perfect" contigs). An exhaustive search across all contigs for a group of clones that are among the candidate clones and are neighbouring clones in a contig can pinpoint the TP clones among the candidate clones. If the marker is present only once in a genome (single copy), only one set of clones should be TP clones. A discrimination score can be assigned to each set of clones and the set of clones with the maximum score is inferred as the most likely TP clones associated with that marker. Because the search is exhaustive and no sole deterministic solution is available for an unbalanced multiple dimensional pooling design, this is an exhaustive search or brute force algorithm for an optimization problem [10,11].

Of 1,384 SNP markers mapped to the *Ae. tauschii *D genome physical map, the contig location of 55 markers had been verified by PCR screening in the previous study [9]. This data set was used to test the new clone deconvolution algorithm. Three different versions of Phase I *Ae. tauschii *contig assemblies were generated with different assembly stringencies and end merges, Assembly 1, Assembly 1.1, and Assembly 2, and these assemblies were used in clone deconvolution for comparison of the "perfectness" of the assemblies (Table 1). Assembly 1 was initiated at 1×10^-15 ^followed by DQing and contig end-to-end merging, and generated 11,852 contigs. The DQing is a process of gradually eliminating questionable clones (Q-clones) by the DQer module in the FPC software [12,13]. The number of contigs was further reduced to 7,447 in Assembly 1.1 by relaxing the conditions of contig end-to-end merges. Assembly 2 is an initial assembly at a higher stringency of 1×10^-60 ^with 17,832 contigs.

**Table 1:** Clone deconvolution of 55 SNP markers mapped on the genetic map of Ae. tauschii chromosome 2D with the 5-D clone pooling strategy.

FPC assembly*	No. of contigs	No. of markers	No. of TP markers	No. of F+ markers	No. of markers without solution	Recall	Precision
1.1	7,447	55	48	4	3	0.87	0.91

1	11,852	55	45	8	2	0.82	0.85

2	17,832	55	8	27	20	0.15	0.23

* (1) Assembly 1: The assembly started at 1×10^-15^, followed by step-wise DQing down to 1×10^-41^. The clones resolved by the DQing process were put back into singletons. The singleton-to-contig end merging was performed at 1×10^-20^. The contig end-to-end merging (requiring a minimum of a 2-clone overlap at each end) at 1×10^-25 ^and at 1×10^-15 ^was performed last. (2) Assembly 1.1: Assembly 1 was subjected to a contig merge. The contig end-to-end merging (requiring only one clone overlap at each end) was performed starting at 1×10^-25 ^and stepping down to the level of 1×10^-15^. (3) Assembly 2: The clones were assembled into contigs at 1×10^-60^.

To evaluate the accuracy of the clone deconvolution algorithm, two performance metrics, recall and precision, were used. The recall is defined as the number of TP markers deconvoluted by the algorithm divided by the total number of markers analyzed, and the precision is defined as the number of TP markers divided by the total number of markers with solutions deconvoluted by the algorithm. The TP markers are those for which TP clones were assigned by the algorithm. Different versions of contig maps resulted in significantly different deconvolution results. In Assembly 1.1, 48 (87%) out of 55 markers were successfully associated with TP clones (0.87 recall) with a precision of 91% (Table 1). In Assembly 2, only 15% of markers were correctly assigned to TP clones. Therefore, a relatively "perfect" contig map is a prerequisite for clone deconvolution. In our example, Assembly 1.1 approximated the "perfect" assembly the best.

**Figure 2:**
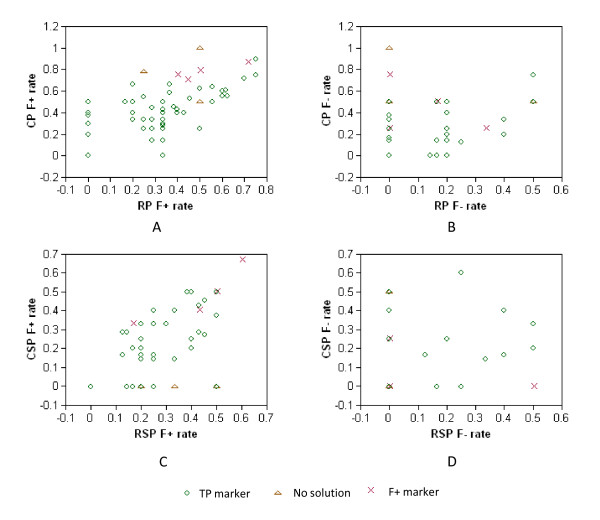
**False positive (F+) pool hit rate and false negative (F-) pool hit rate with clone deconvolution**. (A) Clone row pool (RP) and column pool (CP) F+ rate; (B) RP and CP F- rate; (C) Row super pool (RSP) and column super pool (CSP) F+ rate; and (D) RSP and CSP F- rate. The F+ rate of a pool for a marker is calculated as F+ hits divided by observed pool hits, and the F- rate of a pool for a marker as F- pool hits divided by true pool hits. Based on a given list of true positive BAC clones associated with a marker, true pool hits of CP, RP, CSP and RSP can be reversely obtained from information extracted from clone names. If a true pool hit is not found in the observed pool hits, the hit is F-. However, if an observed pool hit does not exist in the true pool hits, the hit is F+.

In the analysis using the Assembly 1.1 contig map, 3 markers were found without solutions, and 4 markers were assigned to false positive (F+) clones. The seven failed deconvolutions had two primary reasons: (a) low genome coverage in CP or RP (4 markers with < 4× genome equivalent coverage), and (b) F+ and false negative (F-) screening results in CP pools and RP pools (Figure 2). F- pool hits are mostly caused by low genome coverage of pools. F+ pool hits are likely related to either data clustering by the Illumina GoldenGate assay or failures of the Illumina GoldenGate assay. Only one out of 7 failed markers was due to plate super pool deficiency (Figure 2). Obviously, reasonable increase of pool coverage will considerably reduce F- hits and increase the success rate and precision of clone deconvolution.

### Software implementation

**Figure 3:**
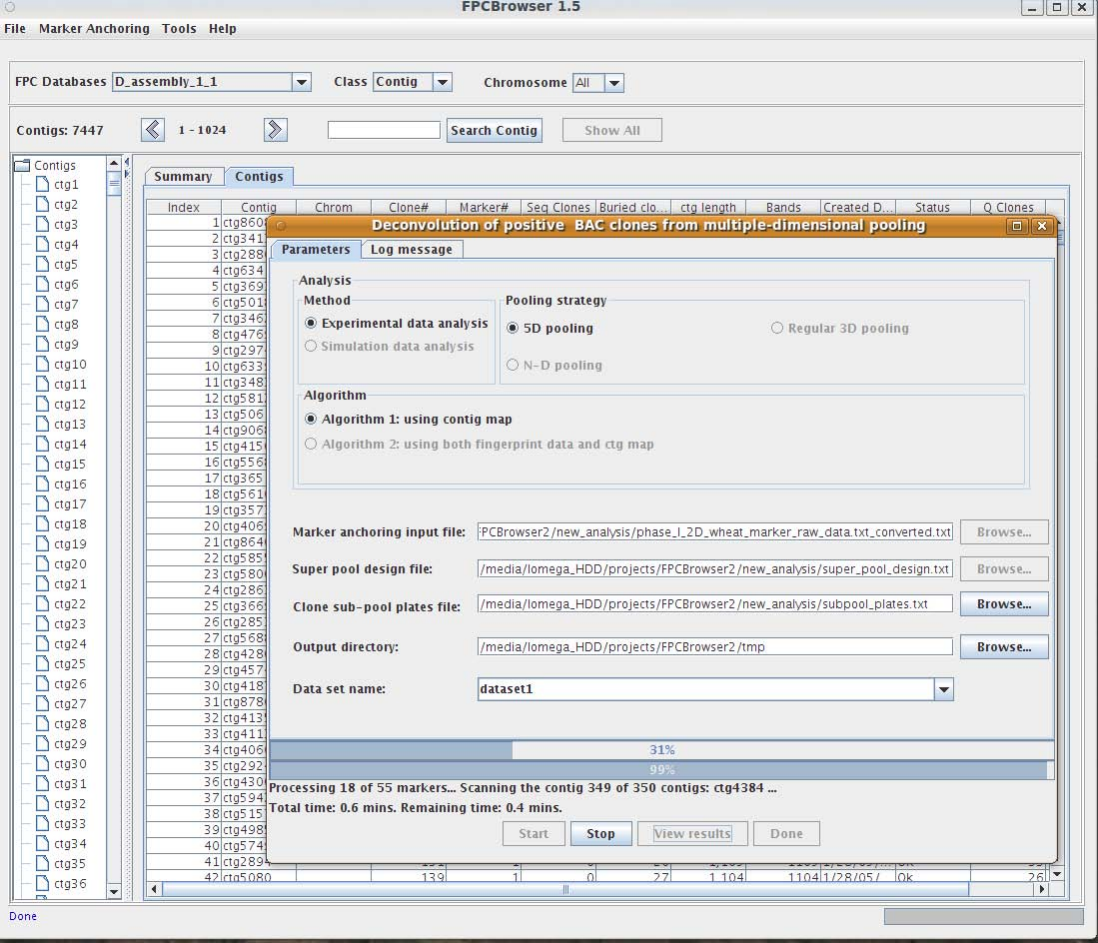
**Screen shot of the FPCBrowser software with the clone deconvolution module running**.

The clone deconvolution algorithm developed for the 5-D clone pooling strategy has been integrated into a newly developed tool, FPCBrowser, a Java-written, platform-dependant and GUI-based software tool (Figure 3). FPCBrowser was initially designed as a portable physical map viewer to comprehensively view FPC contig maps and related information such as clones, fingerprints, and markers in multiple platforms. A Java-based relational database, HSQLDB [14], was adopted in FPCBrowser to store all source data of a FPC contig map and the fingerprints of BAC clones in a physical mapping project, which are also required in clone deconvolution, as well as the analysis results of clone deconvolution. A program module of the clone deconvolution was appended to FPCBrowser for pooling data analysis (Figure 3).

For clone deconvolution, FPCBrowser needs a relatively "perfect" contig map as input (*.fpc, an output file from the FPC software), a 2-D super pool matrix design file and a formatted pool hit file based on the GoldenGate genotyping assay (see details in the FPCBrowser user's guide at http://avena.pw.usda.gov/wheatD/fpcbrowser.shtml) [15]. The clone deconvolution module generates as output a summary file, an anchored positive marker file used for convenient conversion to an ACE file to merge markers into a FPC contig map, and a deconvolution result file for each marker for further manipulation. A Java tool for Ace file conversion directly from the result file is also available in the FPCBrowser package.

Performance of this module depends on several major factors, such as the genome coverage of pools, the number of contigs and contig lengths. When the pooling data of 55 SNP markers and Assembly 1.1 of the contig map were used (Table 1), only 2.1 minutes were needed by a desktop computer (Asus P6T, Intel core i7 920, 12 GB of RAM, and an Ubuntu Linux 9.04 64 bit operating system) to execute the operation. The executable binary version of FPCBrowser is freely available at http://avena.pw.usda.gov/wheatD/fpcbrowser.shtml [15].

## Discussion

The new implementation of the 5-D clone pooling strategy employs the GoldenGate genotyping assay to screen BAC clones in 4 types of pools, RSP, CSP, RP and CP (four dimensions), which replace PCR screening and verification for positive plates in RSP and CSP and for positive clones in RP and CP in the previous implementation [9]. The RP and CP pools are further divided into sub-pools with 1~2× genome coverage to reduce the number of false positive clones in clone deconvolution. Although the total number of screened pools increases, this does not significantly raise the cost for the high-throughput GoldenGate genotyping assay. The fifth dimension is the information about overlapping BAC clones in a contig map which is used to detect and verify truly positive clones among a pool of candidate clones at the intersections of the preceding four dimensions. A clone deconvolution algorithm and corresponding software FPCBrowser have been developed for this purpose. This new implementation provides a high-throughput and low cost approach to BAC library screening and deconvolution of clone pools, and tremendously reduces work load otherwise required for PCR screening and verification of a large number of pools [6-9]. In a test with 55 SNP markers previously associated with *Ae. tauschii *contigs via the GoldenGate assay and manual PCR [9], this implementation yielded a 87% success rate with 91% precision. Some markers resulted in F+ or had no solutions compared to the previous implementation [9]. Improving contig assembly and increasing genome coverage of pools can reduce no-solution and F+ markers.

A "perfect" contig map and adequate pool coverage are two critical components for the clone deconvolution algorithm. Although contig assembly never results in an absolutely perfect contig map because of assembly errors, substandard fingerprinting, chimeric clones, and other reasons, relatively "perfect" contigs can be obtained by an appropriate contig assembly strategy. As long as truly positive clones associated with the marker are overlapping each other in a contig, the contig map for that marker is "perfect", irrespective of the status of the rest of the contig. In addition, if the markers used have been mapped on a genetic map, mapping data can be used for the verification of pool deconvolution. Collocation of markers in a single contig and on a genetic map can validate deconvolution. If a contig is anchored with only a single marker, additional PCR verification of deconvolution may be prudent.

The test data showed that low genome coverage of pools is another reason for F+ anchoring of clones or the inability to place a BAC clone on the physical map (no-solution) resulting in low precision contig anchoring. Although the average coverage of RP and CP was 8.5× in our test data set of 55 SNP markers (Table 2), the actual genome coverage for some of the markers was still low (less than 2×). If a better contig map and higher actual genome coverage were used, a deconvolution success rate higher than the 87% achieved here can be expected.

**Table 2:** BAC libraries and clones used in the 5-D clone pooling design

Library code	Cloning site	Vector	No. of clones	No. of plates	Genome coverage	No. of clones in a row sub-pool	No. of clones in a column sub-pool
RI	EcoRI	pECBAC1	54,144	141	1.6×	3,384	2,256

HB	BamHI	pECBAC1	59,904	156	1.6×	3,744	2,496

BB	BamHI	pCLD04541	76,800	200	1.9×	4,800	3,200

HI	HindIII	pECBAC1	59,904	156	1.8×	3,744	2,496

HI	HindIII	pCLD0451	52,224	136	1.6×	3,264	2,176

Total			302,976	789	8.5×	18,936	12,624

Note: only 14 column pools in the HindIII-pECBAC1 library was used in Illumina GoldenGate genotyping.

## Conclusions

A new implementation of 5-D BAC clone pooling strategy employing both the GoldenGate assay to screen BAC pools and the use of previously assembled BAC contigs is suggested. The implementation is shown to be a high-throughput, low cost, rapid, and feasible approach to screening BAC libraries and anchoring of BAC clones and contigs on genetic maps. The software FPCBrowser with the integrated clone deconvolution algorithm has been developed and is downloadable at http://avena.pw.usda.gov/wheatD/fpcbrowser.shtml [15].

## Methods

### Improved 5-D clone pooling strategy

As described in [9], a conventional 3-D grid pooling is used as part of our algorithm. This pooling strategy includes three different types of pools: plate pools (PP), clone row pools (RP) and clone column pools (CP). For PP, DNAs of 384 clones present in a plate are pooled. To make the screening of PP more efficient, the plate pools are further pooled as a 2-D array. In this 2-D array, PPs are pooled into plate row super-pools (RSP) and plate column super-pools (CSP). For RP and CP screening, the entire stack of 384-well plates is subdivided into N smaller pools (sub-pools) with 1~2× genome coverage. For RP screening, a total of 16 clones × N row pools are generated for each row. Similarly for CP, a total of 24 × N column pools are generated for each column. The four types of pools (RSP, CSP, RP, and CP) are screened with Illumina GoldenGate genotyping techniques [9] in this new implementation of the pooling strategy. Details of DNA pooling methods, Illumina GoldenGate genotyping and scoring of genotyping results were described in [9].

### *Ae. tauschii *Phase I contig maps and BAC screening for 55 SNP markers

Of 302,976 *Ae. tauschii *clones contained in BAC and BiBAC libraries [16], a total of 270,720 were fingerprinted and automatically edited with GenoProfiler [17], and 199,190 were ultimately used for assembly [18,19] with FPC [12,13]. Using different stringencies and end merges, eight Phase I contig maps were generated [18,19]. These draft maps are available at http://wheatdb.ucdavis.edu [19].

All of the 302,976 *Ae. tauschii *clones (789 384-well plates) (Table 2) were pooled into 789 plate pools (each pool containing 384 clones). Plate pools were arranged into a 2-D array, consisting of 27 rows and 30 columns (See Table S1 in Additional file 1). This generated 57 super-pools (27 RSPs, each containing 11,520 clones and 30 CSPs, each containing 10,368 clones), which decreased the number of plate pools for screening to 7%. A total of 190 clone pools, consisting of 80 RPs (5 sub-pools each row, one sub-pool per library) and 110 CPs (5 sub-pools each column, one column sub-pool per library) were generated across all 789 plates. Each clone row sub-pool contained 3,264-4,800 clones and each clone column sub-pool contains 2,176-3,200 clones (Table 2). DNA samples of a total of 217 pools were generated. The pools were screened with Illumina GoldenGate genotyping techniques [9] for 1,384 SNP markers which have been mapped on the *Ae. tauschii *D genome genetic map [20]. Among them, 55 SNP markers mapped on chromosome 2D genetic and physical maps and verified by PCR assay [9] were utilized as test data.

### Algorithm of clone deconvolution using a contig map

Theoretically the 5-D clone pooling is an unbalanced design because a truly positive clone (a well in a plate) for a molecular marker cannot be uniquely determined from the four types of positive pool hits. Positive plate hits are obtained as intersections of positive plate RSP and plate CSP in the 2-D super pool array (Figure 1 and Table S1 in Additional file 1). Candidates of positive clones are at intersections of the positive plates and positive clone RPs and clone CPs. Truly positive intersections must be distinguished from false positives (F+), and such F+ must be removed. For example, pools of clones equivalent to 4× genome coverage are screened with a marker. Four true positive (TP) clones are therefore expected in the pools. Assuming that these clones are in different plates, different rows, and different columns within a plate, there would be a maximum of 4 TP intersections among a total of 256 experimental intersections generating 256 candidate clones. If all clones in a specific row or column of all stack of plates are divided into 4 sub-pools with 1× genome coverage, we can get 4 RPs of 1× genome coverage in a row and 4 CPs of 1× genome coverage in a column, and 1 row pool hit in each sub-row-pool and 1 column pool hit in each sub-column-pool. The total number of candidate intersections will be only (4×4×1×1)×4 = 64. This improved clone pooling strategy for clone row and column pools can dramatically reduce the number of F+ clones. However, 60 of the 64 clones are still F+ clones. A large number of dedicated PCR [9] or extra pools [6-8] are required to eliminate the F+ clones and detect the TP clones. Rather than using PCR, we use information about clone overlaps in the existing contig maps to find the TP clones among the candidates.

**Figure 4:**
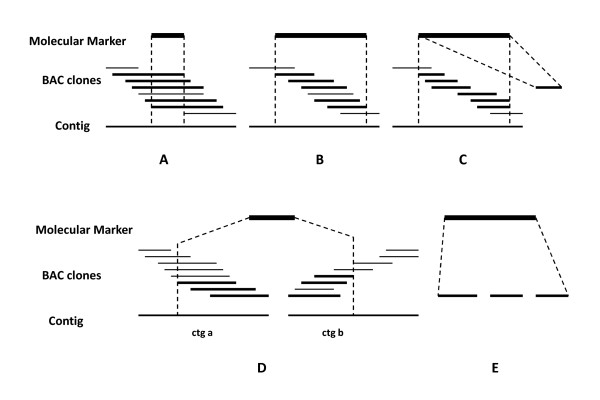
**True positive (TP) clones associated with a molecular marker in a contig map**. Several scenarios were encountered when clones were screened by PCR[9]. (A) All TP clones associated with a marker shared a DNA fragment and had therefore a spanning or inclusion relations. (B) No common DNA fragment was observed in all TP clones associated with a marker but two or more contiguous clones had fingerprint overlaps. (C), (D), and (E) All TP clones associated with a marker were grouped to more than one contig (D), or a contig and singleton (C), or all TP clones were singletons (E). In scenarios C, D, and E, some of the TP clones were usually located at the end of one contig and the rest were located at the end of another contig, or were singletons. The cause of these scenarios was the high stringency used in contig assembly that resulted in two separated contigs or in a contig and a singleton.

Clone deconvolution for a marker identifies TP clones in the population of candidate clones suggested by CSP, RSP, RP, and CP hits. The basic idea is that the TP clones are a subpopulation of candidate clones that have a unique characteristic: they share part of their fingerprint and therefore must be neighbours in a single contig within a well-assembled or a "perfect" contig map. A perfect contig map is almost impossible because of imperfect fingerprints and assembly errors. Here we treat a contig map for a marker to be "perfect" if all TP clones associated with a marker are neighbours within a single contig, irrespective of the veracity of the rest of the contig. Clones should have a spanning relation or inclusion relation (Figure 4A) or at least a simple overlapping relation between each other (Figure 4B). Herein we define that clone **a **and **b **are said to span clone **c **if and only if **f(c) **is contained in the union of **f(a) **and **f(b)**, where **f(a)**, **f(b) **and **f(c) **are fingerprints of clone **a**, **b **and **c**, respectively. Clone **a **is contained in clone **b **if and only if **f(a) **belongs to **f(b)**. Using these relations it is possible to search exhaustively for neighbouring clones among the candidate clones. The search results are often not unique and a discrimination score must be applied to infer an optimal solution. If a search generates no result, the deconvolution has no solution.

In practice, contigs are assembled in several steps, including initial assembly at a high strigency, DQing questionable clones at an increasing stringency, and end-to-end merging and singleton-to-contig end merging at decreasingly lower stringencies. In the contig map assembled at an initial stringency, TP clones for a marker may be scattered among multiple contigs, and/or singletons (Figures 4C, 4D and 4E). Through several merges, a relatively "perfect" contig map can be expected. Using this "perfect" contig map, a computational algorithm was designed as follow:

(1) For each marker, find all positive candidate clones **C **at the intersections of the four types of positive pools (Figure 1).

(2) Search all contigs in the contig map and in each contig find a clone subset **C_1 _**which belongs to **C **if any.

(3) If the size of **C_1 _**(the number of clones) for a contig ≥ 2, find a clone subset **C_2 _**in which clones have spanning or inclusion relation, or overlap each other.

(4) If the size of **C_2 _**for a contig ≥ 2, calculate the number of F- clones (FN). F- clones are the clones which are located between left-most position and right-most position of **C_2 _**in a contig but are not included in **C**.

(5) Calculate a discrimination score for each of all clones sets of **C_2_**: Score = 0.8*NC/MC + 0.2 * (1-FN/NC), where NC is the number of clones in **C_2_**; MC is the maximum number of clones in sets of **C_2_**, i.e., the number of clones in the top set of **C_2 _**after sorting by the number of clones.

(6) Sort all clone sets of **C_2 _**by the score of **C_2 _**in a descending order. Choose the clone set with the highest score. If the score ≤ 0, no solution is found. If there are two or more clone sets which have the same scores, non-unique solutions are obtained for further manual verification.

## Abbreviations

BAC: bacterial artificial chromosome; FPC: fingerprint contig; PCR: polymerase chain reaction; SNP: single nucleotide polymorphism; PP: plate pool; SP: super pool; CSP: column super pool; RSP: row super pool; CP: clone column pool; RP: clone row pool; 5-D: five dimensional; 6-D: six dimensional; TP: true positive; F+: false positive; F-: false negative.

## Authors' contributions

FMY, MCL, ODA and JD planned the work. FMY, MCL and JD developed the clone deconvolution algorithm. FMY designed and implemented the FPCBrowser software. MCL prepared DNAs for Illumina genotyping. FMY and MCL performed data analysis. KX and KRD constructed BAC pools. FMY and JD drafted the manuscript. All authors read and approved the final draft of the manuscript.

## Supplementary Material

Additional file 1 **Table S1**. The matrix design of the super pools of plates. This matrix is used to make super pools of plate DNA and shows row and column coordinates of plates for clone deconvolution. Click here for file

## References

[B1] Barillot E Lacroix B Cohen D Theoretical analysis of library screening using a N-dimensional pooling strategy Nucleic Acids Res 1991 19 22 6241 6247 10.1093/nar/19.22.6241 1956784 PMC329134

[B2] Bruno WJ Knill E Balding DJ Bruce DC Doggett NA Sawhill WW Stallings RL Whittaker CC Torney DC Efficient pooling designs for library screening Genomics 1995 26 1 21 30 10.1016/0888-7543(95)80078-Z 7782082

[B3] Shoemaker RC Grant D Olson T Warren WC Wing R Yu Y Kim H Cregan P Joseph B Futrell-Griggs M Microsatellite discovery from BAC end sequences and genetic mapping to anchor the soybean physical and genetic maps Genome 2008 51 4 294 302 10.1139/G08-010 18356965

[B4] Gardiner J Schroeder S Polacco ML Sanchez-Villeda H Fang Z Morgante M Landewe T Fengler K Useche F Hanafey M Anchoring 9,371 maize expressed sequence tagged unigenes to the bacterial artificial chromosome contig map by two-dimensional overgo hybridization Plant Physiol 2004 134 4 1317 1326 10.1104/pp.103.034538 15020742 PMC419808

[B5] Romanov MN Price JA Dodgson JB Integration of animal linkage and BAC contig maps using overgo hybridization Cytogenet Genome Res 2003 102 1-4 277 281 10.1159/000075763 14970717

[B6] Klein PE Klein RR Cartinhour SW Ulanch PE Dong J Obert JA Morishige DT Schlueter SD Childs KL Ale M A high-throughput AFLP-based method for constructing integrated genetic and physical maps: progress toward a sorghum genome map Genome Res 2000 10 6 789 807 10.1101/gr.10.6.789 10854411 PMC310885

[B7] Yim YS Moak P Sanchez-Villeda H Musket TA Close P Klein PE Mullet JE McMullen MD Fang Z Schaeffer ML A BAC pooling strategy combined with PCR-based screenings in a large, highly repetitive genome enables integration of the maize genetic and physical maps BMC Genomics 2007 8 47 10.1186/1471-2164-8-47 17291341 PMC1821331

[B8] Wu X Zhong G Findley SD Cregan P Stacey G Nguyen HT Genetic marker anchoring by six-dimensional pools for development of a soybean physical map BMC Genomics 2008 9 28 10.1186/1471-2164-9-28 18211698 PMC2259328

[B9] Luo MC Xu K Ma Y Deal KR Nicolet CM Dvorak J A high-throughput strategy for screening of bacterial artificial chromosome libraries and anchoring of clones on a genetic map constructed with single nucleotide polymorphisms BMC Genomics 2009 10 28 10.1186/1471-2164-10-28 19149906 PMC2647554

[B10] Wu Y Liu L Close TJ Lonardi S Deconvoluting BAC-gene relationships using a physical map J Bioinform Comput Biol 2008 6 3 603 622 10.1142/S0219720008003564 18574865

[B11] Jones NC Pevzner PA An introduction to Bioinformatics Algorithms 2004 London, England: The MIT Press

[B12] Soderlund C Humphray S Dunham A French L Contigs built with fingerprints, markers, and FPC V4.7 Genome Res 2000 10 11 1772 1787 10.1101/gr.GR-1375R 11076862 PMC310962

[B13] Soderlund C Longden I Mott R FPC: a system for building contigs from restriction fingerprinted clones Comput Appl Biosci 1997 13 5 523 535 9367125 10.1093/bioinformatics/13.5.523

[B14] HSQLDB http://hsqldb.org/

[B15] FPCBrowser http://avena.pw.usda.gov/wheatD/fpcbrowser.shtml

[B16] Xu Z Deal K Li W Covaleda L Cheng Y Dvorak J Luo M Gill B Anderson O Zhang H Construction and characterization of five large-insert BAC and BIBAC libraries of Aegilops tauschii, the diploid donor of the wheat D genome Plant and Animal Genome X 2002 101

[B17] You FM Luo MC Gu YQ Lazo GR Deal K Dvorak J Anderson OD GenoProfiler: batch processing of high-throughput capillary fingerprinting data Bioinformatics 2007 23 2 240 242 10.1093/bioinformatics/btl494 17018534

[B18] Luo MC Tomas C Deal KR You FM Anderson OD Gu YG Li W Kuraparthy V Gill BS McGuire PE Construction of contigs of Ae. tauchii genomic DNA fragments cloned in BAC and BiBAC vectors10th International wheat Genet Symp: 2003; Paestum, Italy 2003 293 296

[B19] WheatDB http://wheatdb.ucdavis.edu

[B20] Luo MC Deal KR Akhunov ED Akhunova AR Anderson OD Anderson JA Blake N Clegg MT Coleman-Derr D Conley EJ Genome comparisons reveal a dominant mechanism of chromosome number reduction in grasses and accelerated genome evolution in Triticeae Proc Natl Acad Sci USA 2009 106 37 15780 15785 10.1073/pnas.0908195106 19717446 PMC2747195

